# Chromatic and spatial image statistics predict infants’ visual preferences and adults’ aesthetic preferences for art

**DOI:** 10.1167/jov.23.8.2

**Published:** 2023-08-01

**Authors:** Philip McAdams, Megan Chambers, Jenny M. Bosten, Alice E. Skelton, Anna Franklin

**Affiliations:** 1 The Sussex Colour Group & Baby Lab, The School of Psychology, University of Sussex, Brighton, UK; 2 The Sussex Colour Group & Baby Lab, The School of Psychology, University of Sussex, Brighton, UK; 3 Sussex Vision Lab, The School of Psychology, University of Sussex, Brighton, UK; 4 The Sussex Colour Group & Baby Lab, The School of Psychology, University of Sussex, Brighton, UK; 5 The Sussex Colour Group & Baby Lab, The School of Psychology, University of Sussex, Brighton, UK

**Keywords:** art, aesthetics, image statistics, infant, development, color, spatial vision

## Abstract

Aesthetics has been characterized as a triadic interaction of perceptual, emotional, and conceptual neural systems (e.g., Chatterjee & Vartanian, 2014). There has been much empirical effort to identify the visual features that contribute to the perceptual component of this triad (e.g., Mather, 2020). Here, we measured infants’ visual preferences and adults’ aesthetic preferences for 40 of van Gogh's landscape paintings and investigated the contribution of the chromatic and spatial image statistics of the art to infants’ and adults’ responses. We found that infants’ and adults’ responses were significantly related: infants looked longer at the art that the adults found more pleasant. We also found that our combination of chromatic and spatial image statistics could account for around two thirds of the variance in infant looking and adult pleasantness ratings. The amount of variation in the luminance and saturation of the art's pixels contributed to both infants’ visual preferences and adults’ aesthetic preferences, potentially identifying two “perceptual primitives” of aesthetics that can be traced back to early sensory biases in infancy. We also identified important differences in the types of image statistics that predict infants’ and adults’ responses. We discuss the findings in relation to theories of aesthetics, natural scene statistics, and infant vision and perception.

## Introduction

Ever since Fechner (1876), the field of experimental aesthetics has sought to identify the importance of visual features in how people perceive, create, and evaluate aesthetic objects such as visual art (e.g., Palmer, Schloss, & Sammartino, 2013). Although recent neuroaesthetic models propose that aesthetic experience emerges from a triadic interaction of sensory-motor, emotion-valuation, and meaning-knowledge neural systems (e.g., Chatterjee & Vartanian, 2014), there has been much focus in experimental and computational aesthetics on identifying the visual features that are important to the sensory component of this triad. Advances in image processing combined with psychophysics or computational approaches, such as machine learning, have led to the identification of various image features that appear to characterize visual art and that also contribute to people's aesthetic experience (e.g., Brachmann & Redies, 2017). For example, image statistics such as edge density (e.g., Redies, Brachmann, & Wagemans, 2017), edge orientation entropy (the distribution of oriented luminance edges across an image: e.g., Redies et al., 2017), symmetry (e.g., Jahanian, Vishwanathan, & Allebach, 2015), fractality and self-similarity (e.g., Taylor, Micolich, & Jonas, 1999; Amirshahi, Koch, Denzler, & Redies, 2012; Redies, Amirshahi, Koch, & Denzler, 2012), the luminance distribution (e.g., Graham & Field, 2007), certain color statistics (e.g., Leykin & Cutzu, 2003; Nascimento et al., 2017), and Fourier spectral properties (the relative contribution of the spatial frequencies of luminance changes across an image; e.g., Graham & Field, 2008; Redies, Hasenstein, & Denzler, 2008) have all been identified as having an important role in visual art. Models that draw on these image properties can predict a significant amount of the variance in people's preferences for visual art (e.g., up to 75% of the variance in Mather, 2020).

This “perceptual” approach, which focuses on the role of visual features and image statistics in aesthetics, has provided insight into the production and appreciation of art. It has also provided further evidence of the types of information and statistics that the visual system extracts from complex images. The similarities between the image statistics of art and natural scenes (e.g., Graham & Redies, 2010; Montagner, Linhares, Vilarigues, & Nascimento, 2016) have provided further support for the notion that the visual system is optimized to efficiently encode natural scenes (e.g., Simoncelli & Olshausen, 2001). However, the relative importance of visual features and image statistics in aesthetics has been debated. The notion of a set of universal “perceptual primitives” for aesthetics has been challenged (e.g., Brachmann & Redies, 2017), with individual differences (e.g., McManus, Cook, & Hunt, 2010) and cultural variation (e.g., Bode, Helmy, & Bertamini, 2017) noted, and the role of semantic content (e.g., Vessel & Rubin, 2010) and self-identity (e.g., Vessel, Starr, G. G., & Rubin, 2012) emphasized. As suggested by the field of neuroaesthetics (e.g., Chatterjee & Vartanian, 2014), emotion, valuation, knowledge, and meaning are also important components of the aesthetics of visual art.

### Developmental aesthetics

One approach, that could prove useful for understanding the relative contributions and interaction of the visual-sensory, emotion-valuation, and meaning-knowledge components of aesthetics is to investigate responses to visual art at various stages in human development (e.g., Göksun, Kranjec, & Chatterjee, 2014). For example, whereas young infants are undeniably able, on some level, to extract meaning and respond emotionally to visual stimuli (e.g., DeLoache & LoBue, 2009), there is also evidence that infants respond in a more bottom-up manner to visual stimuli, with less top-down influence of experience and knowledge than in adults—the influence of processes such as self-identity and semantics are minimized at such a young age (e.g., Pomaranski, Hayes, Kwon, Henderson, & Oakes, 2021). Because of infants’ greater reliance on bottom-up visual processing, investigating infants’ responses to visual art might therefore contribute to an understanding of the potential “perceptual primitives” of art and aesthetics (Göksun et al., 2014).

Decades of developmental science, dating back to the pioneering studies of Fantz (1958), have established that young infants look longer at visual stimuli with certain features. For example, when shown simple visual stimuli, young infants look longer at (i.e., have “visual preferences” for) high-contrast edges (Banks & Ginsburg, 1985), curved contours (Fantz & Miranda, 1975), and vertical symmetry (Bornstein, Ferdinandsen, & Gross, 1981). Note, infants’ visual preferences are not necessarily “aesthetic”: infants look longer at one stimulus over another for many reasons such as visibility, complexity, novelty, familiarity, and potential for learning (e.g., Houston-Price & Nakai, 2004). However, there is evidence that some aesthetic responses in adults can be partially traced back to simple sensory visual preferences seen in infancy. For example, infants look longer at faces that adults rate as attractive than faces rated as unattractive (e.g., see Damon, Mottier, Méary, & Pascalis, 2017, for a review). In addition, when colors are saturated enough for infants to be able to detect them, infants look longer at colors the more adults prefer them, explaining almost half of the variance in adult color preference (Skelton & Franklin, 2020). Infants’ looking times also predict adults’ preferences for moving dot patterns (Mottier, 2020).

The similarity between infants’ visual preferences and adults’ aesthetic responses for basic visual stimuli raises the question of whether similar relationships are found for visual art. However, despite the potential for infant studies to contribute to our understanding of the role of perception in visual art, very few studies have investigated infants’ visual responses to art. In one study, infants had a visual preference for a selection of the paintings of Picasso over those of Monet, and this preference persisted even when viewed in black and white, or when blurred or distorted (Cacchione, Möhring, & Bertin, 2011). Another study found that six- to 10-month-old infants have a visual preference and adults have an aesthetic preference for art in its original form over art that has been modified to reduce complexity and contrast; and from this it was argued that complexity and contrast are “biologically driven” features contributing to the appreciation of art (Krentz & Earl, 2013). However, a third study potentially challenges the notion of a biological basis of the appreciation of art and found no relationship between infant looking times and adult aesthetic preference for a selection of eight portrait paintings or eight landscape paintings (Mottier, 2020).

### Infant vision and image statistics

Aside from the question of the extent to which adult aesthetic responses to art can be traced back to early sensory biases in infancy, investigating infants’ visual preferences to art can also provide insight into infant vision and visual development more generally. Although decades of research have built an excellent understanding of infant vision for basic visual features such as contrast sensitivity (e.g., Norcia, Tyler, & Hamer, 1990), color (e.g., Skelton, Maule, & Franklin, 2022; Skelton, Franklin, & Bosten, 2023), orientation (e.g., Atkinson, Hood, Wattam-Bell, Anker, & Tricklebank, 1988), and motion (e.g., Banton, Dobkins, & Bertenthal, 2001), there is much less understanding of how infants use these basic visual features to perceive more complex images and scenes. There has been little consideration of whether infants can extract and perceive various image statistics in complex images and scenes, or how this develops throughout childhood. One developmental study found that sensitivity to the Fourier spectral slope does not mature until 10 years of age (Ellemberg, Hansen, & Johnson, 2012). Two other studies suggested some sensitivity of infant perception to natural texture statistics (Balas, Saville, & Schmidt, 2018; Balas & Woods, 2014), and another study found that infants’ sensitivity to color at four to six months old aligns with the typical distribution of chromaticities in natural scenes (Skelton et al., 2023). A recent study suggested that at eight months old, search times for targets increase as the difference in the amount of entropy in the target and background increases (Schlegelmilch & Wertz, 2022). However, there is much more to understand about what image statistics are perceived by the immature visual systems of infants, and the role that these image statistics have in infant perception of complex images and scenes. Addressing these issues is an important part of understanding infant perception, cognition, and behavior; and also has implications for understanding the role of experience in visual development and the role of experience in the optimization tuning of the visual system to efficiently represent natural scenes (e.g., Skelton et al., 2022; Skelton et al., 2023).

### The current study

The current study investigates infants’ visual preferences and adults’ aesthetic preferences for art, compares the similarity of infants’ and adults' responses, and also uses image analysis to investigate the contribution of image statistics to the responses from both groups. Infant looking times and adult judgements of pleasantness (e.g., Cupchik & Gebotys, 1990) are measured for pairs of images of 40 landscape paintings of the Dutch post-impressionist artist Vincent van Gogh. We chose the dimension of “pleasantness” as a measure of adult aesthetics because it is prototypical of positive emotions (Schindler et al., 2017) and is representative of the superordinate hedonic value fundamental to aesthetic evaluations (Berlyne, 1974; Cupchik & Gebotys, 1990). Van Gogh's paintings have been subjected to image analysis previously (e.g., Abry, Wendt, & Jaffard, 2013; Aragón, Naumis, Bai, Tôrres, & Maini, 2006; Berezhnoy, Postma, & van den Herik, 2007; Rigau, Feixas, M., & Sbert, 2008), and an unpublished study suggests that spatial image properties, such as lacunarity (the “gappiness” of an image), entropy, and fractal dimension, can account for the variation in adult preference for his landscape paintings (McAdams & Benson, 2019). Here, our image analysis extracts a set of spatial and chromatic image statistics for each painting. The spatial statistics that we analyze were chosen on the basis of the previous literature on image statistics and adult aesthetics (e.g., Berman et al., 2014; Grebenkina, Brachmann, Bertamini, Kaduhm, & Redies, 2018; Redies, Grebenkina, Mohseni, Kaduhm, & Dobel, 2020; see Brachmann & Redies, 2017, for a review). We compute these spatial statistics for both the luminance and the chromatic components of the image (see also Mather, 2020). The chromatic statistics are partly chosen on the basis of prior research with adults (e.g., Berman et al., 2014; Nakauchi & Tamura, 2022). However, unlike previous research we define colors in a biologically plausible color space that specifies colors in terms of their activation of the two cardinal neural subsystems that underpin color vision (MacLeod & Boynton, 1979), and we also present stimuli on color-calibrated displays. We additionally include chromatic image statistics that are known to characterize natural scenes, such as the extent to which the pixel colors are distributed along the blue-yellow color appearance dimension that relates to color variation in natural scenes (Bosten, Beer, & MacLeod, 2015).

We conduct a correlation to identify the relationship between infant looking times and adult pleasantness ratings, and conduct two forms of regression (backwards linear and partial least squares) to identify the contribution of spatial and chromatic image statistics to infants’ and adults’ responses to art. By investigating the similarity of adults’ pleasantness ratings of van Gogh landscapes and infants’ visual preferences for those paintings, and by establishing the contribution of chromatic and spatial image statistics to adults’ and infants’ responses, we aim to establish the extent to which adults’ aesthetic appreciation of art can be traced back to visual preferences in infancy and whether certain image properties are good candidates for being considered “perceptual primitives” of aesthetics. We also aim to further our understanding of how young infants perceive complex images, identify the types of image statistics that infants can extract, and explore whether infants’ visual preferences are driven by image statistics characteristic of natural scenes.

## Method

### Participants

Twenty-four infants aged between 18 and 40 weeks old (*M* = 26.4 weeks, *SD* = 6.6 weeks, 12 male) took part. An additional four babies were tested, but three were excluded because of general fussiness that prevented them from completing the trials, and one was excluded because of experimenter error. All infants were full term, weighed over 2500 g at birth, had no known neurological or visual conditions, and their parents reported no family history of color vision deficiency. Infants were recruited via social media and given a Baby Lab t-shirt for taking part in the study. There were 20 adult participants aged 18 to 43 years (*M* = 26.5 years, *SD* = 7.7 years, six male), with normal or corrected-to-normal vision, recruited via opportunity sampling from the University of Sussex student and staff body. Adult participants were art naïve, and compensated for their time at payment equivalent to the UK national minimum wage. Adult participants had normal color vision as assessed by the Ishihara pseudoisochromatic plates test (38 plate edition, Ishihara, 1936). Written informed consent was obtained from adult participants and infants’ caregivers; the study conforms to the tenets of the Declaration of Helsinki (but without preregistration), and ethical approval was granted by the University of Sussex Sciences & Technology Cross-Schools Research Ethics Committee (ER/AES31/27) and from the European Research Council Executive Agency.

### Stimuli

**Figure 1.**
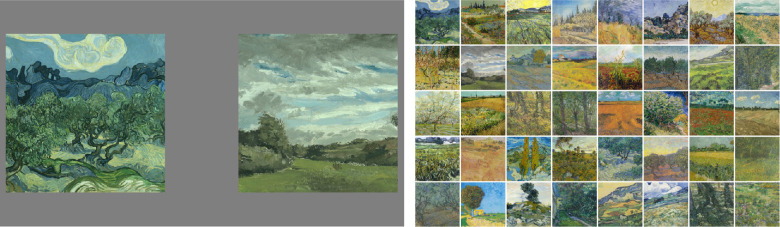
Stimuli were shown paired on a gray background (left) and were sampled from a set of 40 cropped and square digital versions of van Gogh landscapes (right).

Stimuli were 40 high resolution images of landscape oil paintings by Dutch Post-Impressionist artist, Vincent van Gogh, downloaded from the Google Art Project (Google Arts & Culture, 2022) and Wikimedia Commons (Wikimedia Commons, 2022) (see Supplementary Information). Landscapes with no human figures were chosen (see Figure 1). Each image was cropped to the largest central square, and re-scaled to 550 × 550 pixels using bicubic interpolation. Stimuli subtended a visual angle of 14.35° when shown as pairs with their inner edges 2.5° to the left and right of the center point of the screen. Stimuli were displayed on iPad models (Apple, Cupertino, CA) with a screen resolution of 1280 × 720. To render the stimuli equivalently on the various iPad models, we converted the RGB images to LMS, using the Stockman, MacLeod and Johnson (1993) cone fundamentals, via a set of RGB radiance spectra for an arbitrary display. We then calculated hue angle, saturation, and luminance in a version of the MacLeod Boynton (1979) chromaticity diagram that used these fundamentals, and scaled saturation so that all pixels in all images would be in gamut for all the iPad models we used. We then converted back to LMS and then to RGB for display on the particular iPad model used by each participant using a transformation based on the spectra of the three primaries of that iPad model measured using a SpectraScan Spectroradiometer PR-655 (Photo Research Inc., Chatsworth, CA, USA), as in Tang et al. (2022). The neutral gray background on which the stimuli and attention getter were presented was metameric with equal energy white (L/(L + M) = 0.70, S/(L + M) = 1.0). The iPads were set to maximum brightness, and stimuli were presented via a Microsoft PowerPoint (Microsoft Inc., Redmond, WA, USA) slideshow, 1280 × 720 pixels in dimension.

### Image analysis

**Figure 2.**
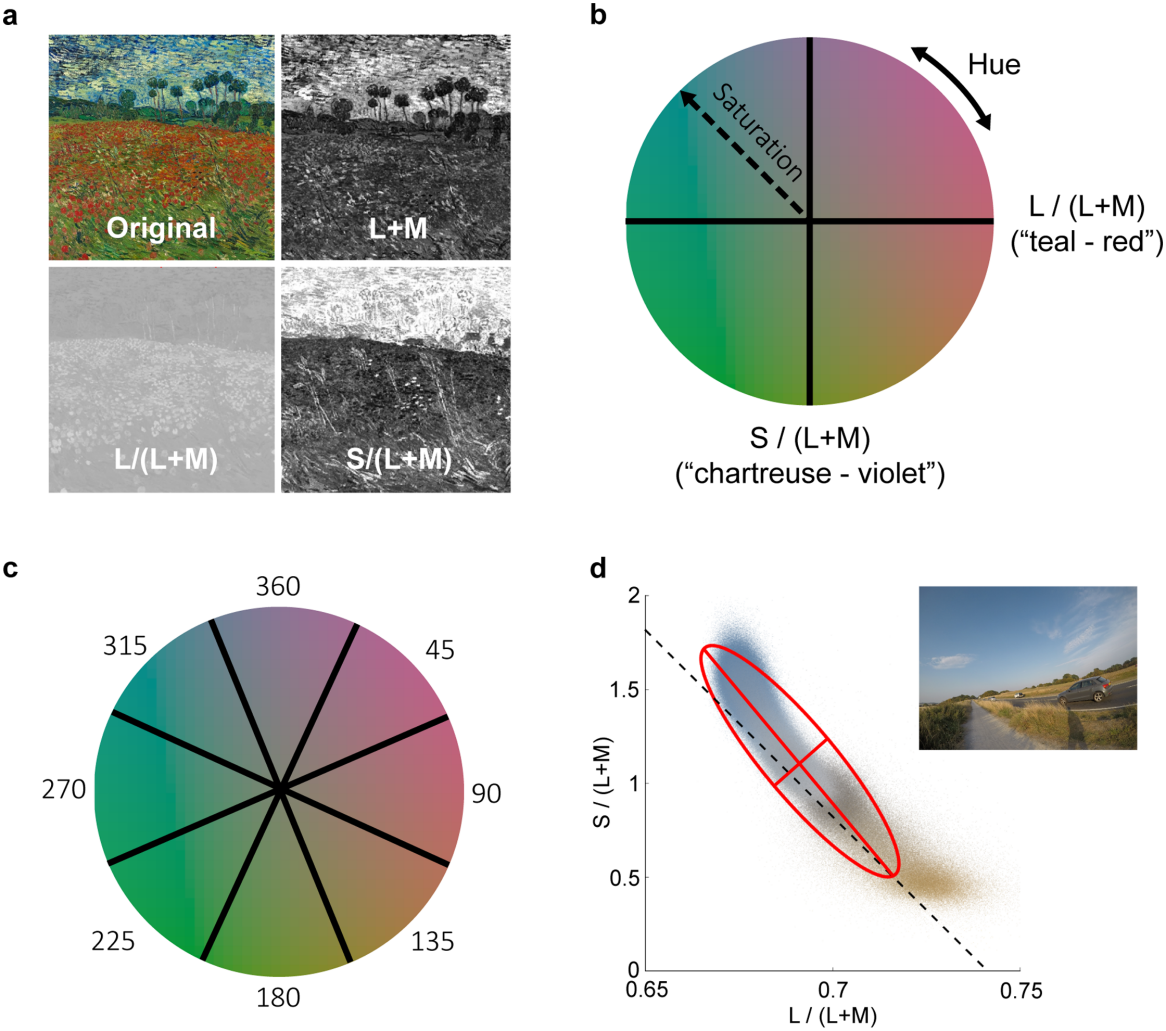
Image statistics were defined for the chromatic and luminance dimensions of the MacLeod Boynton chromaticity diagram: (a) A van Gogh landscape shown in its original form and decomposed for luminance (L + M), teal-red (L/(L + M)), and chartreuse-violet (S/(L + M)). (b) The MacLeod Boynton chromaticity diagram indicating teal-red (L/(L + M)) and chartreuse-violet (S/(L + M)) dimensions, as well as the dimensions of hue and saturation. (c) The MacLeod Boynton chromaticity diagram was divided into eight hue segments of 45°, the numbers refer to the mean hue angle of each segment. (d) The chromaticities of all pixels in the natural scene that appears in the top right-hand corner are plotted in a version of the MacLeod Boynton chromaticity diagram. The “natural chromatic axis,” which roughly connects unique blue and yellow and is close to the locus of natural daylights (see Bosten et al., 2015), is shown by the dashed line. An ellipse has been fit to the distribution of chromaticities, and the axes of the ellipse used to calculate “natural chromatic elongation” (when plotted in the normalized version of the MacLeod Boynton chromaticity diagram) are shown.

Image analyses were conducted using bespoke in-house algorithms (e.g., Skelton et al., 2021; Bosten et al., 2015), built-in MATLAB functions (The MathWorks Inc., 2021), and openly available algorithms (Berman et al., 2014; Mather, 2018; Redies et al., 2017). The majority of the image statistics were computed by converting the RGB image to LMS using the Stockman, MacLeod, and Johnson (1993) cone fundamentals. LMS tristimulus values were used to calculate chromaticity coordinates along the two “cardinal” dimensions of the MacLeod Boynton chromaticity diagram (see Figures 2a and 2b): L/(L + M) and S/(L + M), representing the cone-opponent “teal-red” and “chartreuse-violet” axes of color vision that correspond to the retinogeniculate neural pathways (MacLeod & Boynton, 1979); and L + M, representing luminance. A few image statistics were computed either by converting the RGB image to greyscale using a luma transform (Redies et al., 2017), or to CIELUV color space (CIE, 1986, see below). To reduce the impact of chromatic noise present in very dark pixels, dark pixels with L + M < 0.00045 (using cone fundamentals with 1 nm resolution) were excluded from the calculation of image statistics with the dimensions of saturation, hue, L/(L + M), or S/(L + M).

### Chromatic image statistics

There were three sets of chromatic image statistics. The first analyzed the mean and standard deviation of the image pixel values on the two chromatic axes of the MacLeod Boynton chromaticity diagram, the luminance dimension, and saturation as defined in this color space (radial distance from the equal energy white point, see Figure 2b). We chose to analyze the biologically meaningful chromatic axes of the MacLeod Boynton chromaticity diagram as prior work has established that preference for single colors can be modelled using the cone-opponent channels (e.g., Hurlbert & Ling, 2007). Using this chromaticity diagram to define chromatic axes also enabled us to quantify the natural chromatic distribution of the images (see the third set of measures below) in a way consistent with previous literature (Bosten et al., 2015). For consistency, we calculated saturation in the MacLeod Boynton chromaticity diagram rather than an approximately perceptually uniform color space such as CIELUV (CIE, 1986); however, we note that saturation in the MacLeod-Boynton chromaticity diagram and CIELUV chroma are highly correlated for our image set (*r* = 0.833, *p* < 0.001). To get a measure of the amount of chromatic contrast in the image that captures the perceptual similarity of the image colors, we also computed the root mean square of the Euclidian distance between each pixel in the image from the average chromaticity of all pixels in the image in the perceptually uniform CIE u*v* chromaticity plane (by converting the RGB image to CIE XYZ (CIE, 1932) and then to CIE L*u*v* (CIE, 1986).

The second set of chromatic statistics aimed to capture the hues present in the images. To quantify the hue, the MacLeod Boynton chromaticity diagram was divided into 8 radial hue segments centered on equal energy white (see Figure 2c), and for each image we computed the percentage of pixels that belonged to each hue segment and the mean saturation of the pixels in each hue segment. Pixels with a very low saturation (<0.05) were filtered out to prevent a large number of off-white pixels on one or the other side of the chosen white point being arbitrarily assigned to a particular hue bin. No published studies have investigated the relationship between these hue measures and adult preferences; yet, as hue is an important perceptual dimension of color (Burns & Shepp, 1988), we felt that it warranted investigation. The third set of chromatic image statistics aimed to capture the extent to which the chromaticities of the pixels form a distribution that aligns with the chromatic distributions typically found in natural scenes, which are elongated along an oblique axis in the MacLeod Boynton chromaticity diagram roughly between unique blue and unique yellow and close to the locus of natural daylights (e.g., Bosten et al., 2015; Mollon, 2006). One prior study has found that adult preference peaks for Mondrian stimuli that vary in their chromaticity along this “blue-yellow” axis compared to other color axes (Juricevic, Land, Wilkins, & Webster, 2010). To compute these measures, standard deviation ellipses were fit to the chromaticity distribution of the pixels in each image, in a version of the MacLeod-Boynton chromaticity diagram where the variances along the x and y axes were each normalized (Bosten et al., 2015). In this space, any ellipse with a major axis oriented along the negative diagonal is biased in roughly a blue-yellow direction. We calculated the log of the ratio of the length of the ellipse axis oriented along the negative diagonal (called the “natural chromatic axis” hereafter, see Figure 2d) and the length of the ellipse's orthogonal axis. This is a measure of the extent to which the chromaticity distribution is elongated along the natural chromatic axis (we term this measure “natural chromatic elongation,” Bosten et al. (2015) referred to this as the “axis ratio”). A value of 0 indicates that the pixels form a circular distribution in the color space with no bias in any particular direction, and positive values indicates greater elongation along the natural chromatic axis than its orthogonal one, whilst negative values indicate greater elongation along the orthogonal axis than the natural chromatic axis. We also computed the angle of the major axis of the ellipse in the original un-normalized version of the MacLeod Boynton chromaticity diagram (without variances normalized along the x- and y-axes—Bosten et al. (2015) referred to this as the “ellipse angle”), because this angle also relates to the “naturalness” of the color distribution.

### Spatial image statistics

A number of image statistics capturing spatial structure were computed for luminance (L + M), S/(L + M), and L/(L + M) chromatic dimensions (of the MacLeod Boynton chromaticity diagram): spectral slope, fractal dimension, lacunarity, and several types of edge density. Spectral slope measures the relative contributions of various spatial frequencies to the structure of an image as a whole (e.g., Graham & Redies, 2010). Low spatial frequencies represent large scale structures, such as the horizon separating sky and ground in a landscape image; whereas, high spatial frequencies represent fine detail (e.g., tree leaves). When amplitude is plotted on logarithmic axes against spatial frequency, for natural scenes there is a characteristic negative “spectral slope” of approximately −1 (Billock, 2000). We calculated spectral slope for luminance and both chromatic axes in the MacLeod-Boynton chromaticity diagram.

Fractal dimension measures the degree to which self-repeating patterns across scales fill a space (Mandelbrot, 1982; Spehar, Clifford, Newell, & Taylor, 2003). This quality of self-similarity and rough scale invariance can be found in natural scenes and art (Graham & Redies, 2010), and fractal patterns are present throughout nature (e.g., in a landscape's trees, mountains, and clouds). Fractal dimension is suggested to relate to aesthetic preference (e.g., Hagerhall, Purcell, & Taylor, 2004). Here, we use two box-counting-based fractal dimension measures: one-dimensional (1-D) fractal dimension, which measures the degree to which a 1-D pattern fills a two-dimensional (2-D) plane (Coburn et al., 2019; Moisy, 2022); and 2-D fractal dimension, which measures how a 2-D plane pattern fills a three-dimensional space (Mather, 2018; Graham, 2020). Fractal dimension is related to visual complexity, the higher the value the more intricate and detailed the pattern.

Lacunarity characterizes the spatial distribution of gap sizes across an image and the degree of non-uniformity of an image's texture (Mandelbrot, 1982; Plotnick et al., 1996). An image high in lacunarity has a wide range of gap sizes, high heterogeneity, with low rotational and translational invariance; whereas, a low lacunarity image is mostly homogeneous, with high rotational and translational invariance, and a narrow range of gap sizes (e.g., Newman, Kennedy, Falk, & McKenzie, 2019; Vernon-Carter, Lobato-Calleros, Escarela-Perez, Rodriguez, & Alvarez-Ramirez, 2009). Lacunarity was computed using a gliding box counting technique (Reuter, 2016) for luminance (L + M) only.

Entropy measures the probability of encountering particular intensity level values across an image (e.g., Kersten, 1987). Images low in entropy show predictability or redundancy of the intensity of pixels across an image, whereas images high in entropy show more randomness and unpredictability (Chandler & Field, 2007). Entropy can be used to characterize the texture of an image (Berman et al., 2014) and was calculated here using the MATLAB (The MathWorks Inc., 2021) “entropy” function, based on Shannon entropy.

Edge density, including straight-edge density (edges belonging to lines) and non-straight-edge density (edges belonging to curves) were calculated as described in Berman et al. (2014), with high values indicating more edges. An edge is a luminance or color change representing an object or scene boundary. Edge density provides a measure of the complexity and texture in an image (Berman et al. 2014).

We also computed a number of image statistics from Redies et al. (2012; 2017): Gabor edge density, first- and second-order edge orientation entropy (EOE), and a set of statistics which use the pyramid histograms of oriented luminance gradients (PHOGs).

Gabor edge density was computed as described by Redies et al. (2017). Images were first converted from color to grayscale using the ITU-R-601-2 luma transform (ITU, 2017), which weights the color channels according to their perceived luminosity. Then, images were filtered using a set of 24 oriented Gabor filters representing one full rotation, akin to receptive fields in the human visual system (Olshausen & Field, 1996). The phase and deviation of the Gabor filters were set to one zero crossing in the middle, so the filters would respond maximally to edges in an image (Redies et al., 2017). The orientations of all the edges in an image were determined and then all edge responses were summed (see Redies et al., 2017, for further details).

Edge co-occurrence statistics were also computed using the method and code of Redies et al. (2017). First, the same procedure as for Gabor edge density was performed, then first-order EOE was calculated from the histogram of edge orientations, measuring the distribution of luminance edges across all orientations in an image (Redies et al., 2017): low values indicate that particular orientations predominate in an image, and high values indicate that orientations are represented more equally. Second-order EOE was calculated by a pairwise comparison of all oriented edges, measuring how predictable edge orientations are across an image: low values indicate that edge orientations at a given position can predict the orientations at other positions in an image; whereas, high values indicate edge orientations at one location are less predictive of other edge orientations at other locations.

Finally, we used another set of image statistics developed by Redies et al. (2012), which use PHOGs to measure spatial image properties at different levels of scale. The set included a measure of self-similarity, which compares the HOGs in sub-regions of an image, with the same feature in the whole image; anisotropy, which compares the difference in strength of the HOGs across levels; and complexity, which measures the mean strength of luminance and color changes across the image (Redies et al., 2012). These PHOG measures were calculated for luminance (L + M), S/(L + M), and L/(L + M).

### Design and procedure

Each participant saw a subset of 10 stimuli, and every stimulus in a subset was paired with every other stimulus in that subset. The location of each image in every pair, left or right, was flipped per trial for each participant at random, so that each image was presented either on the left or right nine times, giving 45 pairs of trials in total. The order of the 45 pairs was randomized per participant. For infants, we used an iPad preferential looking procedure developed in-house (Skelton et al., 2021, and described in full here), which enabled us to test remotely during the COVID-19 pandemic. Infants were seated on their caregiver's lap, 30 cm from the iPad screen, in a dimly lit room in the infant's home. Infants were shown a PowerPoint display on their iPads, shared by the experimenter via Zoom (Zoom Video Communications Inc., 2023); and infants viewed this display in full-screen mode with the camera window and any other windows minimized. We recorded the full-screen presentations of the PowerPoint as seen on the infants’ iPads via Zoom and the camera recording of the infants’ faces, and these were recorded as two separate but time-synchronized recordings.

At the start of the infant session, infants completed four calibration trials to aid later coding, where infant looking was fixated centrally, then to the left, and right, and centrally again, using a black and white dynamic attention-getting stimulus. Following this, infants saw the 45 trials of pairs of images, displayed for five seconds each. Between each trial, the attention-getting stimulus was displayed, lasting for varying times as decided by the experimenter, to ensure the infant's attention was centrally located at the start of a trial. After the testing session, infant looking time to each image was manually coded, blind to the experimental condition of each trial, using DataVyu (Datavyu Team, 2014). Fifteen percent of the data was double coded, blind to the condition, giving an inter-rater reliability across stimuli of *r* = 0.86.

For adult participants, the design was identical to that of the infants’ except that there was no inter-trial attention-getter. Adults attended the experiment in-person, in a dimly-lit laboratory room, and were seated 30 cm from an iPad at eye-level. Participants were instructed that their task was to tap the picture they found most pleasant.

## Results

### Comparison of infant looking time and adult pleasantness scores

**Figure 3.**
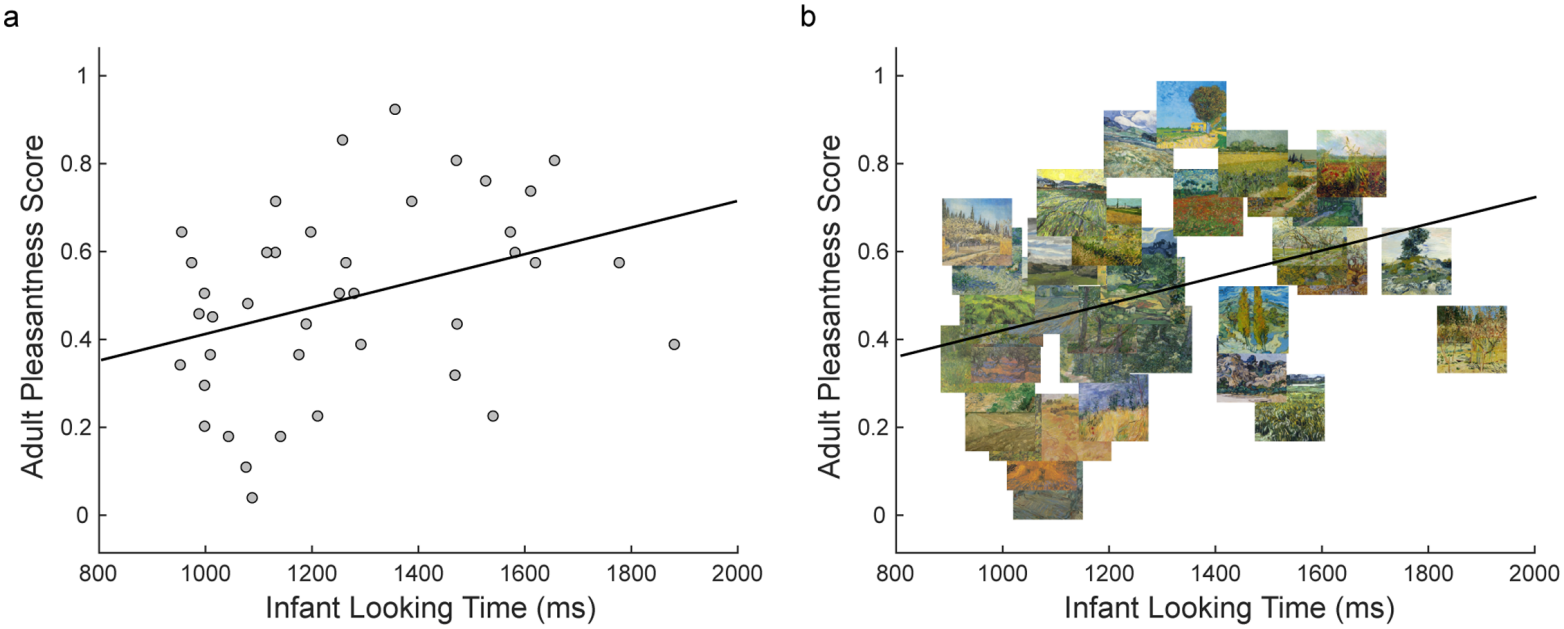
The relationship between infant looking time and adult pleasantness scores (averaged across participants) across the 40 van Gogh images: (a) Data for each image is indicated with a gray circle. (b) Thumbnail plots of each image have been centered on the data point for that image to give insight into the visual characteristics of the relationship.

The average looking time for an infant for each artwork was calculated, averaging across all presentations of that artwork on left and right sides. This average looking time measure was then averaged across infants for each artwork. For adults, an average pleasantness score for each artwork was calculated by averaging the number of times that an image was chosen as the most pleasant in the pair, across trials, for each image and for every adult, and then this measure was averaged across adults. Infant looking time and adult pleasantness scores across the artworks revealed a moderate relationship where infants looked longer at artwork that adults rated as more pleasant, *r* = 0.35, *n* = 40, *p* = 0.025 (see Figure 3).

### Regression analyses

We conducted two types of regression to identify the image statistics that have a linear relationship with looking time or pleasantness scores. Image statistics in art and natural scenes are often correlated because of the content of the image (e.g., trees are likely to be both highly fractal and green). The two regression types that we conduct deal with this collinearity in different ways. First, we conduct multiple linear regressions with multicollinearity analysis, which eliminates variables that are highly related to other variables (Alin, 2010; Fox & Monette, 1992). We include this analysis because it is conventional, although dropping collinear variables from the analysis is not fully satisfactory because it ignores the contribution of those variables. Therefore we also conduct partial least squares regressions (PLSR), as in Mather (2020). PLSR can be used when predictors are collinear (as they often are in art and natural scenes) and combines aspects of principal component analysis and multiple linear regression - it works by generating a component model based on linear combinations of predictor variables (Wold, Martens, & Wold, 1983). In this way, PLSR is a useful tool for finding the optimal combination of image statistics that predict infant looking and adult liking. The PLSR gives a variable importance in the projection (VIP) score for each image statistic, which gives an estimate of the importance of each image statistic for the model (Wold, Johansson, & Cocchi, 1993).

For the multiple regressions, variables were first screened for normality, collinearity, and outliers, using JASP (JASP Team, 2023). Then the image statistics and outcome variables were converted into z-scores to place them all on the same scale and to ensure equal weight in the model fitting (e.g., Mather, 2020). To select variables, we used a backward elimination model, in which all predictors are entered simultaneously, and then removed sequentially if the *p* value of regression coefficient > 0.1 (Dunkler, Plischke, Leffondré, & Heinze, 2014). In this way, redundant variables were removed from the model. Then, to further control for multicollinearity among the image statistics, we calculated variance inflation factors (VIFs), using the collinearity diagnostics function in JASP, which measure how much the variance of an estimated regression coefficient is increased because of collinearity (Fox & Monette, 1992). When there is no multicollinearity, explanatory variables have a VIF equal to 1; if the VIF of an explanatory variable equals 10, then 90% of the variance in that variable can be explained by the other independent variables, and there is multicollinearity present. We removed variables with the highest VIFs, until all VIFs were < 1.25 (i.e., <20% variance explained by other predictors, and indicative of no multicollinearity issues, e.g., Fox & Monette, 1992; Graham, 2003). To corroborate the results, a forced entry regression method was also performed by entering all potential predictor variables into the model, then systematically removing variables that didn't contribute to the model (Field, 2017), then performing the same multicollinearity analysis. The same models for infants and adults were obtained with both the backward regression and forced entry methods.

For the PLSR, variables were also converted into z-scores and the PLSR was conducted using the “plsregress” function in MATLAB (The MathWorks Inc., 2021). To avoid under- or over-fitting, we determined the optimal number of components in our PLSR models by using cross-validation. VIP scores, which are a weighted sum of squares of the PLS-weights calculated from the amount of Y-variance of each PLS component (Wold, Sjöström, & Eriksson, 2001), were also computed (see “plsregress” function in MATLAB). Components with a VIP score greater than 1 are more important than average. The VIP threshold value used in the present study for component selection was 1.25, as Chong and Jun (2005) recommend a value higher than 1 in the case of high correlation among predictor variables.

#### Infant looking time

For infant looking time, all assumptions of normality, linearity, homoscedasticity, and multicollinearity for the multiple linear regression were found to be met after 1 variable was removed due to being an influential outlier: Self-Similarity L/(L + M). The multiple linear regression model significantly predicted infant looking time, *F*(4, 35) = 7.83, *p* < 0.001, adj. *R*^2^ = .412. Four variables added to the prediction, three positive and one negative, which accounted for 41% of the variance in infant looking times (Table 1). The PLSR analysis on infant looking times identified a two-component model of a combination of image statistics that accounted for 70.5% of the variance in infant looking time. Twelve variables had VIP scores above the critical value of 1.25 (Table 2).

**Table 1.** Results of the multiple linear regression for infants (top table) and for adults (bottom table). Negative relationships are indicated with a “−” before the *t* statistic.

Coefficients	Standardized beta	*t*	*p*
Infants			
Entropy L/(L+M)	−0.455	−3.329	.002
Standard Deviation of Luminance	0.262	2.179	.036
Standard Deviation of Saturation	0.278	2.053	.048
Straight-Edge Density S/(L + M)	0.254	1.973	.056
Adults			
Standard Deviation of Saturation	0.404	3.159	.003
Proportion of Green Pixels	0.383	3.040	.005
Straight-Edge Density L/(L + M)	−0.330	−2.653	.012
1D Fractal Dimension L + M	−0.281	−2.417	.039
Standard Deviation of Luminance	0.237	1.856	.072

**Table 2.** Results of the PLSR on infant looking times and adult pleasantness scores: VIP scores for image statistics which contributed to the model (>1.25). Negative relationships are indicated with a “−ve” after the VIP score.

Image statistic	VIP
Infant	
Gabor edge density	1.85
Lacunarity	1.83
Anisotropy S/(L + M)	1.65
SD of luminance	1.65
Entropy L/(L + M)	1.60-ve
Straight-edge density S/(L + M)	1.59
Edge orientation entropy first-order	1.58
Spectral Slope L/(L + M)	1.44
Entropy S/(L + M)	1.44-ve
Edge orientation entropy second-order	1.33
SD of saturation	1.32
Saturation of chartreuse pixels (segment 180 on Figure 2c)	1.27
Adult	
SD of luminance	1.84
SD of saturation	1.76
Entropy L + M	1.69
Straight-edge density L/(L + M)	1.49-ve
Proportion of yellow pixels (segment 135 on Figure 2c)	1.47-ve
Proportion of green pixels (segment 225 on Figure 2c)	1.46
1D fractal dimension L/(L + M)	1.44-ve
1D fractal dimension L + M	1.44-ve
Anisotropy L + M	1.25-ve

#### Adult pleasantness scores

For adult pleasantness scores, all assumptions of normality, linearity, homoscedasticity, and multicollinearity for the multiple linear regression were found to be met after 1 influential outlier was removed: Mean L/(L+M). The regression model significantly predicted adult preference scores, *F*(5, 34) = 7.25, *p* < 0.001, adj. *R*^2^ = .445, with three positive and two negative predictors. The PLSR on adult pleasantness scores identified a 2-component model which accounted for 63.4% of the variance. Nine variables had VIP scores above the critical value of 1.25 (Table 2).

### Additional analyses

To address the possibility of our analysis being underpowered, we conducted two additional analyses. First, we carried out a Bayes factor (BF) analysis, which quantifies the strength of evidence for the alternative hypothesis relative to the null hypothesis (Wagenmakers et al., 2017). A BF_10_ of 3 and above indicates “moderate” evidence for the alternative hypothesis over the null hypothesis (Jeffreys, 1939). Using JASP (JASP Team, 2023), we conducted a Bayesian linear regression, choosing a default prior distribution given that prior knowledge was absent or vague (e.g., see Ly, Verhagen, & Wagenmakers, 2016). We used the Jeffreys-Zellner-Siow prior which uses the Jeffreys prior on sigma and the Zellner-Siow Cauchy prior on the coefficients with an *r*-scale of 0.354 (Consonni, Fouskakis, Liseo, & Ntzoufras, 2018). We found the same coefficients and weightings as in the standard multiple linear regression model, a BF_10_ of 236 for infants, and a BF_10_ of 294 for adults.

Second, we carried out a permutation analysis, which uses random resampling of the observed data as the “data” for inference, breaking the link between predictors and observed outcome but preserving all other aspects of the data (Fisher, 1935). This allows the discovery of the distribution of expected statistical outcomes under the null hypothesis (where there is no link between infant looking time, or adult liking, and the particular images). For the permutation analysis, we reran our PLSR analysis, using MATLAB (The MathWorks Inc., 2021), 10,000 times (e.g., see LaFleur & Greevy, 2009), each time randomly shuffling the infant looking times or adult pleasantness ratings across images, and calculating the optimal number of components using cross-validation per permutation. We generated a reference distribution of variance explained, ranging from 0.28% to 50.94% (*M* = 24.30, *SD* = 8.51) for infants, and from 0.23% to 52.15% (*M* = 24.38, *SD* = 8.57) for adults. Our observed variance explained for infants (70.5%) was more than 5 *SD* higher than the reference distribution mean, and for adults (63.4%), was more than 4 *SD* higher. We also investigated whether the same set of significant predictors was observed in any of the 10,000 permutations; however, the same set never appeared in infant or adult analyses. These results suggest that it is highly unlikely that our observed results occurred randomly.

## Discussion

The current study aimed to identify the contributions of chromatic and spatial image statistics to infants’ visual preferences and adults’ aesthetic preferences for van Gogh landscapes, and to identify how similar infants and adults are in their preferences and in the types of image statistics that contribute to their responses. We found that both infant and adult preferences could be well predicted by the chromatic and spatial image statistics of van Gogh's art. For the multiple regressions, just under a half of the variance in adults’ aesthetic preferences and infants’ visual preferences could be explained by a set of image statistics. The same image statistics were identified as important in the PLSRs, but the fact that collinear predictors could be kept in the model meant that the PLSRs identified several other image statistics that were important and around two thirds of the variance could be explained by the two component models for infants and adults.

Although there was some similarity in the image statistics that contributed to infant and adult preferences, there were also notable differences, explaining the significant but low amount of shared variance (only 13%) between infant looking and adult pleasantness measures. The main similarity was that adults found art more pleasant and infants looked longer the greater the luminance and saturation variation in the art. The amount of entropy, anisotropy, and edges also contributed to both infant looking and adult liking, although with a different direction of relationship or for different color dimensions. For example, whereas adults preferred low anisotropy in the luminance dimension, infants preferred high anisotropy in the chartreuse-violet dimension. Whereas adults found art more pleasant the greater the luminance entropy, infants looked longer at art with less chartreuse-violet and teal-red entropy. Adults also found art more pleasant when there were fewer straight edges defined by the teal-red dimension, whereas infants looked longer at the art the greater the amount of straight edges defined by the chartreuse-violet dimension and the more luminance edges of any kind (straight and non-straight). Hue was important to both adult liking and infant looking, yet the percentage of green and yellow pixels was important for adults, and the saturation of chartreuse pixels was important for infants.

There were also a number of image statistics that predicted adult liking but not infant looking, and vice versa. Adults found art more pleasant the smaller the fractal dimension for luminance and teal-red contrast; however, fractal dimension did not contribute to infant looking. Although the fractal dimension was not important to infants, infants did look longer at art with greater lacunarity, a measure that is related to fractal dimension; and lacunarity did not relate to adults’ liking. The spectral slope of the teal-red dimension positively contributed to infant looking but not adult liking. The orientation of edges was also important for infants but not adults: infants looked longer at art the greater the first-order and second-order edge orientation entropy, indicating a preference for art where edges are more evenly distributed across the range of orientations and where edge orientations across the art are weakly correlated.

These findings have implications for our understanding of how the visual features of art contribute to a mature aesthetic response in adulthood, the notion that there are “perceptual primitives” of aesthetics that can be traced back to simple visual preferences in infancy, and of the role of image statistics in early visual development. We discuss each of these in turn.

### Image statistics and adult aesthetics

We establish that around two thirds of the variance in adults’ pleasantness judgements of van Gogh's landscapes can be accounted for by a set of image statistics. The amount of variance explained here is typically larger than in previous studies that have had different sets of image statistics and modelled aesthetics for different types and genres of art, although some studies have modelled a comparable amount of variance. For example, Mather (2020) analyzed the contribution of spectral slope, entropy, and fractal dimension on luminance, red-green, and blue-yellow axes of the CIELAB color space to adult ratings of beauty, and found that 75% of the variance in adults’ beauty ratings of nudes could be accounted for. For landscapes, only 22% of the variance in beauty could be accounted for by Mather's analysis, with red-green spectral slope, luminance entropy, and blue-yellow entropy being important predictors. The greater amount of variance explained for landscape aesthetics in the current study than in Mather's may be due to the additional image statistics in the current study that were added to the model which were shown to be important such as hue, anisotropy, and variance of luminance and saturation. Another possibility is that the difference is due to the range of landscape paintings that were included: Mather's study included landscape paintings from various artists and genres whereas here we analyzed the aesthetic responses just for van Gogh's landscapes. Van Gogh's art has a particular style that may tend to emphasize certain image statistics, and having a restricted range of art in the stimulus set in the current study might increase the contribution of image statistics because personal preferences for particular artists or genres are minimized. The different aesthetic ratings—pleasantness in the current study and beauty in Mather's—could also potentially account for the differences in variance and important predictors.

Many of the image statistics that predicted adults’ pleasantness judgments in the current study have been identified as contributing to various types of aesthetic judgements in prior research. For example, Mather (2020) established that adults find landscapes with unpredictable luminance differences more beautiful, and we replicate that result for pleasantness judgements here. We, unlike Mather, also find that fractal dimension contributes to adult aesthetics for landscapes. Prior work has identified that fractal dimension predicts aesthetic preference for abstract art, with preference peaking for mid-range fractal dimension and decreasing at low and high values (e.g., Taylor, Spehar, Hagerhall, & Van Donkelaar, 2011). In our study, fractal dimension had a negative relationship to pleasantness, but the fractal dimensions of the landscapes ranged from medium to high, and so the negative relationship is consistent with preference at the mid-range. We also found that anisotropy and edge density predict adult aesthetics, and these image statistics have featured in prior models of aesthetics for various types of art as well (e.g., Hayn-Leichsenring, Lehmann, & Redies, 2017; Sidhu, McDougall, Jalava, & Bodner, 2018).

In the current study, color had a role in infants’ and adults’ responses. Whilst color statistics have previously been found to predict aesthetics of various types of art (e.g., Hayn-Leichsenring et al., 2017; Lyssenko, Redies, & Hayn-Leichsenring, 2016; Sidhu et al., 2018), our novel approach of quantifying the percentage and saturation of pixels falling into eight hue categories enabled us to identify the particular importance of green and yellow hues. We found that the naturalness of the chromatic distribution (as measured by the extent to which the chromatic distribution aligns with the typical chromatic distribution of natural scenes) did not predict adults’ pleasantness judgements. One prior study has found that adult preferences for abstract chromatic Mondrian stimuli peaks for stimuli that contain colors that vary along the “natural” blue-yellow color appearance axis that relates to color variation in natural scenes, compared to other axes in color space (Juricevic et al., 2010). However, we find no evidence for that preference when considering the more multi-colored chromatic distributions of art. One possibility is that this image statistic was made salient in Juricevic et al.’s study because the axis of the chromatic distribution was the only feature to vary in the stimulus set: the relative importance of the image statistic could be reduced when other image statistics are also free to vary. Possibly, the range of chromatic distributions in van Gogh's landscapes also does not have sufficient variability to enable the measure to predict. However, our finding is consistent with those of two prior studies which found that people prefer paintings in their original chromatic distribution, which is more reddish than the natural chromatic axis (Nascimento et al., 2017), and that the color statistics that predict preference for art differ to those of natural scenes (Nakuchi & Tamura, 2022).

In sum, our analyses of adult pleasantness judgements of van Gogh's landscapes support the notion that image statistics contribute to aesthetics, and provide support for the involvement of image statistics identified in prior research as well as identifying the contribution of new ones. However, comparison of our findings to those of other studies also supports the notion that the types of image statistics and their relationships with aesthetics are likely to vary with type and genre of art (e.g., Hayn-Leichsenring et al., 2017; Mather, 2020), how variable the set of stimuli are on each of the image statistic measures, and the type of aesthetic judgement (e.g., Augustin et al., 2012; Lyssenko et al., 2016). We chose to investigate pleasantness judgements here, although further work that explores how our model applies to other types of aesthetic judgements such as interest or beauty could be interesting.

### “Perceptual primitives” of aesthetics

The current study found a significant but relatively small amount of shared variance between infants’ visual preferences and adults’ pleasantness judgements for van Gogh's landscapes. It appears therefore that there is much less similarity in infant looking and adult liking for landscape paintings than for visual stimuli such as color (Skelton & Franklin, 2020), moving dots (Mottier, 2020), and faces (e.g., Damon et al., 2017). One possibility is that the more basic or elemental the visual stimulus, the more bottom-up are the adults’ responses and more similar to infants’ responses. For landscape paintings, there is potentially greater room, than for colors or moving dots, for the role of emotion and knowledge to influence adults’ judgements. Although some of the infants in our sample may not yet perceive pictorial depth cues (see Kavšek et al., 2012, for a review) and, hence, view the landscapes more abstractly, it is theoretically possible that the older infants, who have more experience of different environments and landscapes, might also have emotional and cognitive responses to landscapes. However, these influences are expected to be stronger in adults who have more experience of the world and also more experience of art.

Although the shared variance is small, the current study nevertheless does find a significant relationship between infant looking and adult liking for art landscapes. This contrasts with a prior study that found no relationship between infant looking and adult liking for eight landscape paintings of various artists (Mottier, 2020). One possibility for the difference in results is that Mottier's stimulus set had different artists whereas our stimuli were all from the same artist—Mottier’s stimulus set could give more room for adult judgements to be driven by personal preferences for certain artists. One prior study identified similarity between infants’ and adults’ responses to art, finding that infants look longer at, and adults prefer, original paintings than paintings that have been manipulated to reduce complexity and contrast (Krentz & Earl, 2013). It was argued that a preference for art with high complexity and contrast was “biologically driven.” The current study also identifies that infants’ visual preferences and adults’ aesthetic preferences increase as the contrast in the art increases, with both saturation and luminance variation positively predicting preference. This could point to saturation and luminance contrast being “perceptual primitives” of aesthetics—features of mature aesthetic preference that can be traced back to sensory biases in infancy. However, whether such primitives are biologically driven is contentious. Even a young infant has visual experience of the world, and visual preferences in infancy could well be a result of this experience and even a result of the developing visual system calibrating to the statistical features of natural scenes (e.g., Skelton et al, 2022; Maule, Skelton, & Franklin, 2023).

We also find that edge density, entropy, and anisotropy contribute to both infant and adult measures. However, we do not find strong support for Krentz and Earl's (2013) proposal that preference for complexity drives both infants’ and adults’ responses to art. Although infants’ responses had a positive relationship to anisotropy, in the chartreuse-violet dimension, relating to adult subjective ratings of lower complexity, higher structure, and less interest (Lyssenko et al., 2016); adults’ responses had a negative relationship to anisotropy, in the luminance dimension (a preference for lower variation in the luminance gradients across orientations), relating to prior subjective ratings of more complexity, less structure, and more interest (Lyssenko et al., 2016). However, although adults found van Gogh's landscapes more pleasant the greater the unpredictability, infants looked longer the more predictable they were. The fractal dimension also negatively predicted adults’ pleasantness judgements and did not contribute to infants’ visual preferences. Differences between infants’ and adults’ responses to the complexity of the art could be due to differences in their visual capabilities and the immaturities of infant vision such as poor visual acuity and contrast sensitivity. The “Goldilocks effect” (e.g., Kidd, Piantadosi, & Aslin, 2012) proposes that infants don't prefer greater complexity but a level of complexity that is “just right” for their level of ability. Further research that investigates the relationship of image complexity and infants’ visual preferences, given infants’ levels of visual ability, would be useful.

### Infant vision and image statistics

Beyond the question of the nature of aesthetics, the current study has implications for our understanding of infant vision and scene perception. Whilst there has been decades of research on infants’ visual preferences for basic visual stimuli (e.g., Banks & Ginsburg, 1985; Bornstein et al., 1981; Decarli, Piazza, & Izard, 2023; Fantz, 1958; Fantz & Miranda, 1975; Fantz & Nevis, 1967; Kanazawa, Kitaoka, & Yamaguchi, 2013; Otsuka & Yamaguchi, 2003; Quinn, Brown, & Streppa, 1997; Shuwairi, 2009; Sifre et al., 2018; Spears, 1964), infants’ visual preferences for complex images such as scenes, and their sensitivity to image statistics is poorly understood. The role of image statistics in visual development is important since image statistics have a major role in mature vision, and in object and scene perception, recognition, and categorization in adults (see Geisler, 2008, for a review). The role of image statistics in visual development could also provide insight into when vision is optimized for natural scenes (e.g., Skelton et al., 2023), with implications for understanding the role of experience in the calibration of vision to natural scenes (e.g., Bosten, Coen-Cagli, Franklin, Solomon, & Webster, 2022; Simoncelli & Olshausen, 2001).

Here we show, on the basis of the best fitting PLSR model, that a number of image statistics are relevant to infants’ visual preferences: variance in luminance and saturation, entropy, anisotropy, edge density, first- and second-order edge orientation entropy, lacunarity, spectral slope, and the saturation of certain hues. The influence of some of these image statistics on infant perception has not previously been identified. For example, although prior research has pointed to infants looking longer at curvilinear than straight stimuli (e.g., Fantz & Miranda, 1975), it has not been previously established that infants are sensitive to both the uniformity in the distribution of edge orientations and the relationship of edge orientations across the image. High edge orientation entropy is a characteristic of natural scenes that is typically amplified in art (Redies et al., 2017), and infants’ visual preferences for images with high EOE could well indicate that vision is optimized for certain natural scene statistics even from a young age.

The relationship of infants’ visual preferences to teal-red spectral slope also warrants further investigation. One prior study has suggested that due to immaturities in spatial vision, that children's visual systems aren't optimally sensitive to the spectral slope of natural scenes until 10 years of age, whilst adult sensitivity is greatest for spectral slopes of −1.3 (Ellemberg et al., 2012). In the current study, we find that infants look least at images with a teal-red slope of around −1.3, and that looking time roughly doubles as the slope decreases to −1.0 (a post-hoc Pearson's correlation gave *r* = 0.37 for this relationship). Ellemberg et al. measured sensitivity to the spectral slope of natural scenes by measuring thresholds to a change in the slope of natural images, and showed that for six- and eight-year-olds, their thresholds were equivalent for spectral slopes of −0.7, −1.0, and −1.3. To find in the present study that infants’ visual preferences are sensitive to the spectral slope of art is therefore surprising. One possibility is that the relationship is due to spectral slope correlating with some other feature of the art, rather than it uniquely driving infants’ responses. There is also a question of how spectral slope should be calculated for infants: given infants’ poorer contrast sensitivity and spatial vision, an image that gives a spectral slope of −1.3 for adults would be expected to have a different spectral slope when seen through infants’ blurred vision. We are currently further investigating and modelling the impact that infants’ visual immaturities have on their perception of image statistics for complex images and scenes.

## Conclusion

In conclusion, we find some similarity in how long infants look at van Gogh's landscapes and how pleasant adults find them. We also find that both infants’ and adults’ responses to van Gogh's landscapes can be well explained by the chromatic and spatial image statistics of the art. We find that infants look longer and adults find the art more pleasant as the standard deviation of the saturation and luminance of the art increases, potentially suggesting that greater saturation and luminance contrast are “perceptual primitives” of aesthetics. Other image statistics such as anisotropy and edge density also contributed to both infants’ and adults’ responses to the art. However, the contribution of other image statistics to adult aesthetics cannot similarly be traced back to infants’ visual preferences. Differences in the way image statistics contribute to infants’ and adults’ responses suggest that infants have their own responses to art that are distinct from adult aesthetics. Infants’ responses to art is likely a result of both their visual immaturity and reduced visual experience as well as their relatively immature emotional and cognitive processing. Further research on “developmental aesthetics” is warranted, as is further exploration of the role of image and natural scene statistics during visual development.

## Supplementary Material

Supplement 1
